# Treatment of Advanced Breast Carcinoma with Drostanolone Propionate

**Published:** 1970-07

**Authors:** N. M. Heney

**Affiliations:** Senior House Officer in Surgery, Southmead and Frenchay Hospitals, Bristol


### Treatment of Advanced Breast Carcinoma with Drostanolone Propionate

### A case report by

**N. M. Heney, M.D.**

**Senior House Officer in Surgery, Southmead and Frenchay Hospitals, Bristol**

It is widely accepted that in cases of advanced breast cancer hormonal therapy can result in a temporary remission of symptoms with relief of pain and prolongation of life (Stott, 1969). Steroids, androgens, oestrogens, castration, adrenalectomy and hypophysectomy have all been used with some success, and it is believed now that a change in the hormonal environment of the tumour and its metastases is as likely to produce a remission as any more specific treatment.

Methods of determining if a tumour is hormone dependant have been investigated by Bulbrook (1964) who described a method based on the urinary excretion of steroids. The observation that prolonged use of androgenic hormones caused virilising effects led to the development of 2-alpha-methyldihydrotestosterone propionate or drostanolone propionate (Masteril), which is an androgenic hormone that does not produce significant virilising effects (Blackburn and Childs, 1959).

The clinical response to this hormone of a pre-menopausal patient with painful bone secondaries is described. This was the first occasion on which this drug was used in Britain for human therapy.

### CASE REPORT

Mrs. V. P., a fifty-five year old pre-menopausal lady had a modified left radical mastectomy in June 1965, for a well differentiated mucus secreting carcinoma without axillary gland involvement. She remained well until December 1967 when she developed pain in both legs, and x-rays revealed osteolytic metastases in her pelvis and femora. Treatment with drostanolone propionate 100 mg intra-muscularly every week was commenced in December 1967, and within two weeks her pain had completely disappeared. Pain recurred after five months, in the right leg, and x-rays demonstrated widespread lytic and porotic secondaries in the chest, pelvis, femora and lumbar spine. Bilateral oophorectomy was performed in June 1968 and pain was relieved for ten months. Four months after the oophorectomy, pelvic secondaries, which before had been osteolytic, were noted to be sclerotic. Two months later, in December 1968 her widespread metastases showed an increase in size, and it was thought that the tumour cells had escaped from the effects of the drug. Pain remained under control until, after an attack of influenza in March 1969, she developed severe pain in the right shoulder region, due to a secondary deposit. After bilateral adrenalectomy in May 1969, although her pain was relieved in the immediate post-operative period, she developed left foot drop. Drostanolone propionate, which had been administered without break since December 1967, was stopped at the time of the adrenalectomy.

By July, 1969, although her foot drop had improved, she had developed lumbar and right scapular pain and showed the presence of painless skull secondaries. Drostanolone propionate was re-started, and within two weeks she was pain free in her back, shoulder and leg and there followed further improvement in her foot drop. Chest x-ray revealed barely detectable osteosclerotic metastases. In September 1969 roentogenography failed to show evidence of further skeletal metastases, her foot drop had disappeared and she was able to return to part time machine work. X-ray in January 1970 failed to show an increase in size in the metastatic deposits as compared with May, 1969.

### DISCUSSION

Tagnon (1969) suggests that early in their development, metastatic deposits maintain a strong identity with the parent tissue and during this time are hormone-dependant. Later this identity is lost and the tumour cells become hormone-independent. Deshpande (1967) demonstrated that drostanolone propionate reduced the uptake of oestradiol-17B by tumour cells, whereas Altman and Chayen (1967) suggested that the drug acted by diverting the reduced co-enzyme NADPH into the metabolically wasteful diaphorase system thus limiting the amount of biosynthesis in the carcinoma.

Evidence that the cancer cells were hormone-dependent was suggested by the relief of bone pain after the administration of drostanolone propionate. Although the initial treatment failed to control the growth of secondary deposits, pain was relieved for some months. Oophorectomy caused a temporary osteosclerosis and a ten month remission from pain. After seventeen months of continuous treatment with drostanolone propionate there were no signs of masculinzation. Adrenalectomy relieved pain for two months, by which time there was evidence of further spread of osteolytic lesions. Within two weeks of re-starting drostanolone therapy, pain had again disappeared and the metastases started to become sclerotic.

This case illustrates some interesting features which invite various suggestions.

(i) Drostanolone propionate can produce remission of pain in the presence of active ovaries, though it is specially recommended for the post menopausal patient.

(ii) A trial period on drostanolone propionate suggested that the tumour cells were hormone dependent and is offered as a simple clinical way of determining this fact.

(iii) Virilization was not a noticeable side effect.

(iv) Axillary glands were not involved by tumour at the time of operation, yet distant spread had occurred. This re-inforces the argument in favour of chemotherapeutic "cover" at the time of surgery and drostanolone propionate may prove a useful adjunct to surgery as such an agent.

(v) A febrile illness, such as influenza can produce a period of accelerated growth in bony metastatic deposits. This raises the question of immunology and demands that patients should be closely watched after such an illness.

### ACKNOWLEDGEMENTS

I am indebted to Dr. A. Otlet for permission to report on this patient of his, and to Mr. L. R. Celestin for allowing me to present this case of historical and clinical interest.

### REFERENCES

**Altman, F. P. and J. Chayen** (1967). Proceedings of symposium "The Treatment of Cancer of the Breast". Excerpta Medica Foundation, pp 56-63.

**Blackburn, C. M. and D. S. Childs.** Use of 2-alpha-methyl androstan-17 beta-ol, 3-one (2-alpha-methyl dihydrotestosterone) in the treatment of advanced cancer of the breast (1959). Proceedings of the Staff Meetings of the Mayo Clinic **34,** 113.

**Bulbrook, R. D., J. L. Hayward and B. S. Thomas.** The Relation between the Urinary 17-hydroxycorticosteroids and 11-deoxy-17-oxosteroids and the Fate of Patients after Mastectomy. (1964). Lancet **1,** 945.

**Deshpande, N.** (1967). Proceedings of symposium. "The Treatment of Cancer of the Breast". Excerpta Medica Foundation, pp 44-55.

**Stoll, B. A.** Hormonal Management of Advanced Breast Cancer. (1969). British Medical Journal **2,** 293.

**Tagnon, H. J.** Scientific Basis of Cancer Chemotherapy. (1969). Heinemann, London pp. 66-71.

